# Tympanometry in neonates with normal otoacoustic emissions: measurements and interpretation

**DOI:** 10.1016/S1808-8694(15)30123-3

**Published:** 2015-10-19

**Authors:** Kilza de Arruda Lyra e Silva, Beatriz de Albuquerque C.C. Novaes, Dóris Ruthi Lewis, Renata Mota Mamede Carvallo

**Affiliations:** a Master’s degree, Speech Therapist - HU/UFAL; b Ph.D., Professor, PUC-SP; c Doctor, Professor, PUC-SP; d Doctor, Adjunct Professor - FMUSP. Pontifical Catholic University, São Paulo - PUC-SP

**Keywords:** neonates, middle ear, tympanometry

## Abstract

Tympanometry is used in evaluating middle ear functional conditions. Before six months of age its results may be misleading. High frequency studies aim to provide more valid procedures. **Aim:** To describe and discuss tympanometric measurements and the interpretation in normal hearing neonates at 226, 678 and 1000Hz. **Method:** 110 neonates that were analyzed had normal otoacoustic emissions and no risk for hearing impairment. The age range was 6 to 30 days. Curves were obtained using the GSI-33-II, at the “Divisão de Educação e Reabilitação dos Distúrbios da Comunicação”, São Paulo, in 2004. **Study design:** Clinical prospective. **Results:** There was a balance between single and double peak curves at 226Hz. Most of the curves were asymmetric at 678Hz, and single-peaked at 1000Hz. quantitative measurements showed a significant gender difference in the Equivalent Ear Canal Volume at 226Hz and on the Peak Compensated Static Acoustic Admittance at 1000Hz. The English protocol showed that almost 100% of ears were normal at 678 and 1000Hz. **Conclusion:** 1000Hz yielded superior results for characterizing normality. The English protocol was efficient to reduce the variability of tympanometric measurements. Data from this study may be used as a guide for diagnosis using tympanometry in neonates.

## INTRODUCTION

The aim of early identification and characterization of hearing loss in neonates is to generate the conditions for adequate interventions as early as possible to reduce the negative consequences for the child’s personal and social development. Studies by Yoshinaga-Itano et al.^1^ have suggested that babies diagnosed with hearing loss before age six months had a superior performance in language abilities compared to those in which hearing loss was diagnosed at a later stage.

Audiological assessment uses a test battery that involves joint analysis of results and that should incorporate objective and behavioral measurements and procedures to avoid diagnostic errors.^2^ Early diagnosis of hearing loss requires testing of the middle ear to differentiate sensorineural and conduction hearing loss. This separation is important to identify hearing loss caused by transient conditions in the middle or external ear, and to establish the need for clinical management of middle ear diseases. It may also support the need and timing of monitoring procedures such as Brainstem Auditory Evoked Potential testing (BAEP).^3^

Acoustic immitance testing is a general term that describes the ease or opposition to the flow of sound energy in a given system. This test is a well-proven simple procedure for detecting and classifying middle ear changes, and for assessing the integrity of the peripheral auditory system in children and adults. Acoustic immitance measurements may be classified into dynamic and static testing.^4^ Static measurements assess tympanic membrane complacency, while dynamic measurements are tympanometry and measurement of the estribo muscle reflex.

Tympanometry is an objective test to evaluate middle ear function. It is an easy and simple procedure for detecting and classifying middle ear changes in children and adults; it is, therefore, essential for increased diagnostic efficacy. Clinical testing is routinely done at a 226 Hz probe tone.

Tympanometry in lactating babies aged below six months, done using 226 Hz as the test tone, may not be reliable, as lactating babies with otitis media may reveal an apparently normal tympanogram when using this probe.^5^, ^6^, ^3^ This has led researchers^3^, ^7^, ^8^, ^9^ to investigate high frequency probes (678 or 1000 Hz) in lactating babies, to attain increased reliability.

Different result interpretation criteria may lead to different conclusions, and consequently different diagnostic approaches. Northern and Downs10 have emphasized that details may be lost if we use only the qualitative evaluation of tympanometry. Quantitative criteria, which are influenced by transmission factors in the auditory system, should be added to avoid occasional error and misinterpretation.

Quantification of tympanometric measurements make it possible to develop appropriate guidelines for comparing tympanometric data generated in different clinical units, and to guide sensitivity and specificity data not yet formalized.^11^ Quantitative measurements are used together with qualitative data to characterize tympanograms with greater precision. These features include:^10^, ^11^, ^12^ Peak Compensated Static Acoustic Admittance (Ymt), or height of the tympanogram in the tympanic membrane plane; Equivalent Volume of the External Acoustic Meatus (Vea), or the estimated middle ear air volume; Tympanogram Gradient (TG) and Tympanometric Width (TW), or description of the tympanogram shape close to the peak; and Tympanogram Peak Pressure (TPP), or location of the tympanographic peak along the pressure axis.

Few tympanometric studies have been made of lactating babies under age six months. Furthermore, details of the neonatal middle ear mechanoacoustic properties have not been widely investigated. Both systematic and normative studies are needed to improve the usefulness of tympanometry in auditory diagnosis of neonates.^13^

This study aims to describe and analyze interpretations of the features and measurements obtained in tympanometry done on listening neonates aged between six and 30 days, using a 226, 678 and 1000 Hz trial probe tone. The following features of the tympanogram are described: Characteristics of the Tympanometric Curve, Peak Compensated Static Acoustic Admittance at the Tympanic Membrane, Tympanometric Width, Tympanometric Peak Pressure, Equivalent Volume of the External Acoustic Meatus and the recommended protocol for tympanometry in lactating babies below age four months.^14^

## METHOD

This study was approved by the Research Ethics Committee of the Sao Paulo Catholic University (PUC-SP), protocol number 0011/2004. Neonate caretakers signed a free informed consent form that allows the data to be used in research and in disseminating the results, according to the Resolution 196/96 of the National Health Council.

The investigation included 143 lactating babies born in the Sao Paulo Amparo Maternal Hospital. The results of 110 neonates were used in this study (58 male and 52 female) aged between 6 and 30 days.

Neonate inclusion criteria were term birth, no pre-, peri- or postnatal complications that could cause auditory alterations, not being neonates at risk according to the criteria presented in the Joint Committee on Infant Hearing, 15 and passing the TOAE screening test. The clinical history was taken from parents or caretakers to establish if the neonates met inclusion criteria. Otoscopy was not done on the neonates, as the presence of TOAEs was considered as a sign of normalcy.

TOAE testing and tympanometry were done in the neonatal auditory screening unit of the Education and Rehabilitation of Communication Disorders Division (DERDIC), PUC-SP, in sound-proofed rooms equipped according to the equipment manufacturer’s technical requirements.

Passing or failing the TOAE screening test involved positive responses as those that had a general reproducibility ≥ 50%, presence of TOAEs in four consecutive frequency bands, a signal to noise ratio ≥ 3 dB NPS in the first two bands, and ≥ 6 dB NPS in the three last bands, inclusion of 4000 Hz testing and probe stability ≥ 75%.^16^, ^17^

TOAE testing was done using an Otodynamics ILO 92 cochlear emission analyzer and tympanometry was done using an automatic Grason Stadler GSI 33 (version II) middle ear analyzer with a graphic printer. The intensity of the equipment probe trial tone was 85 dB NPS at 226 Hz, 80 dB NPS at 678 Hz and 75 dB NPS at 1000 Hz.

Tympanometry was done by inserting a probe in the ear of neonates to apply variable pressure between +200 daPa to 400 daPa at 50 daPa/s. This test was done with trial probe tones at 226, 678 and 1000 Hz. Neonates underwent the test with no rigid presentation of the probe tone. Most of the tests were started at 226 Hz followed by 678 and 1000 Hz. Neonates were place in their parent’s lap, in a tranquil state or sleeping naturally, with pauses for breast feeding and hygiene as needed. Choice of the ear to begin the exam was done randomly, depending on the position of the neonate on the lap of his or her caretaker. If the left arm of the caretaker supported the neonate, the test was started on the left ear (which was readily accessible). If otherwise, the test was started on the right ear. The test was repeated if the curve required confirmation.

The following quantitative measurements were obtained and assessed in tympanograms using the probe at 226 and 1000Hz, as follows: Peak Compensated Static Acoustic Admittance at the Tympanic Membrane (Ymt - difference between the admittance peak and admittance at + 200 daPa); Tympanometric Width (TW - pressure difference between two points on the curve where admittance is half the peak admittance); Tympanometric Peak Pressure (TPP). At 226 Hz the Equivalent Volume of the External Acoustic Meatus (Vea) was analyzed. The highest peak were measured if a double peak was present.

Analysis of variance (ANOVA) was the statistical procedure for calculating the significance of quantitative measurements Vea, Ymt, TPP and TW at a 95% significance level (p < 0.05). Two factors were used, namely the ear (right/left) and the sex (male/female) and measurements were repeated in one factor (Ear).

The recommended protocol for tympanometry in lactating babies below age four months14 was used for tympanogram probe results at 678 and 1000 Hz. This protocol considers a tympanogram as normal if Ymt > 0 and TPP > -200 daPa, and abnormal if Ymt <= 0 or TPP < -200 daPa.

## RESULTS

There were 220 tympanograms for each probe frequency. Findings of the 660 tympanograms revealed tympanometric curves as follows: single peak (PU) and double peak (PD) on the 226 Hz trial probe tone; PU, asymmetrical (AS), and inverted (I) types in the 678 Hz trial probe tone; and PU, AS, and I types in the 1000 Hz trial probe tone. Table 1 shows these results, presenting the total value and percentages found in tympanograms for each curve type in each trial probe tone.

**Table 1:** Tympanometric curves related to probe frequency in 110 neonates (58 male and 52 female) in both ears.

Type	226 Hz		678 Hz		1000 Hz	
	N	%	N	%	N	%
PU	105	47,7	56	25,4	156	70,9
PD	115	52,3				
AS			148	67,3	62	28,2
I			16	7,3	2	0,9

Total	220	100,0	220	100,0	220	100,0

A second investigation was to identify the occurrence of the same curve type in both ears of a neonate for each probe frequency. Table 2 shows the results of this investigation, separated into the total and percentage value for neonates presenting similar (equal) and different curve types in both ears and for the three probes.

**Table 2:** Total equal and different curve types in both ears in 110 male and female neonates.

Type	226 Hz		678 Hz		1000 Hz	
	N	%	N	%	N	%
Equal						
PU	36	32,7	16	14,5	68	61,8
PD	41	37,3				
AS			60	54,5	21	19,1
I			2	1,8	1	0,9
Different	33	30,0	32	29,1	20	18,2

Total	110	100,0	110	100,0	110	100,0

A comparison was made between equal curve types found with 226 Hz and 1000 Hz probes, illustrated in Table 3. There was a low occurrence of similar curve type configurations in both ears and frequencies.

**Table 3:** Joint comparison of curve types for 226 and 1000 Hz probes in both ears in 110 male and female neonates.

226 Hz / 1000 Hz	N	%
PU / PU	23	20,9
PD / PU	26	23,6
Other	61	55,5

Total	110	100,0

Quantitative variables obtained in single peak tympanograms using 1000 Hz probes, and single and double peak tympanograms using 226 Hz probes were also evaluated, besides the analysis of curve type occurrence. Results obtained with the 678 Hz probe were not assessed because only 25.4% of these tympanograms revealed a single peak.

Table 4 presents the p-values obtained from the ANOVA. A gender significance effect for Vea (p=0.026) was noted for the 226 Hz probe. There was a significant gender effect in Ymt (p=0.000) for the 1000 Hz probe, which analyzed data obtained from PU type curve tympanograms. Other measurements did not present significance effects according to the ear or the sex.

**Table 4:** Results of the statistical analysis - p-values obtained by ANOVA.

Probe	Variable	Equality of means test in both ears	Equality of means test in both sexes
		p-value	p-value
226 Hz	Vea	0,100	0,026
	TPP	0,538	0,716
	Ymt	0,080	0,144
	TW	0,058	0,989
1000 Hz	TPP	0,392	0,690
	Ymt	0,924	0,000
	TW	0,817	0,958

The means, standard deviations and percentiles (5, 50 e 95) were calculated based on ANOVA results. Results on Table 5 for the 226 Hz probe and on Table 6 for the 1000 Hz probe are shown separately when there was a significant gender and/or ear effect, and shown jointly when there was no ear and/or gender significance effect.

**Table 5:** Quantitative measurements of 220 tympanograms with the 226 Hz probe.

Measurement	Sex	N	Mean	Standard deviation	5	Percentiles 50	95
V_ea_	Female	104	0,64	0,01	0,50	0,60	0,80
	Male	116	0,68	0,01	0,50	0,60	0,82
TPP		220	-8,93	31,40	-60,00	-5,00	39,75
Y_mt_		220	0,75	0,22	0,40	0,80	1,10

TW		220	115,05	40,60	45,25	115,00	184,75

**Table 6:** Quantitative measurements of 220 tympanograms with the 1000 Hz probe.

Measurement	Sex	N	Mean	Standard deviation	5	Percentiles 50	95
TPP		156	-3,46	44,21	-90,00	0,00	61,50
Y_mt_	Female	72	0,90	0,47	0,30	0,75	1,94
	Male	84	1,36	0,75	0,50	1,20	3,15
TW		156	99,49	24,73	60,00	100,00	145,00

Another form of interpreting tympanograms is that based on the protocol proposed by Sutton et al.^14^ This protocol classifies tympanograms as normal or abnormal. Our results based on this analysis is shown on Table 7 and reveals a high occurrence of normalcy for both probes.

**Table 7:** Result of the tympanometric analysis based on Sutton et al.’s14 protocol in 220 ears using test probe tones at 678 and 1000Hz.

	678 Hz		1000 Hz	
	N	%	N	%
Normal	208	94,5	217	98,6
Abnormal	12	4,5	3	1,4

Total	220	100,0	220	100,0

## DISCUSSION

All of the neonates were considered as normal or listeners, having passed neonatal auditory screening. Detection of TOAEs requires clean external ears and no tympanic perforation for stimulus transmission to the cochlea and reverse transmission of external acoustic meatus emissions.^18^, ^19^

Tympanometry measurements in the sample neonates showed tympanograms with PU and PD curve types based on the 226 Hz trial probe tone, and PU, AS and I curve types in 678 Hz and 1000 Hz trial probe tones. Of these curve types, only the PU type was framed within the traditional Jerger20 classification as similar to type “A” in his definition. There is, however, no evident reason for the other tympanometric curve types in neonates. The PD type tympanogram, similar to the PU type, is also considered as indicating normalcy in neonates, as reported in various published studies.^21^, ^22^, ^23^ There is no consensus about the AS and I curve types as indicators of normalcy.

For the 226 Hz probe our findings showed a balance between PU (49.1%) and PD (50.9%) curve types in 220 ears. This tympanometric configuration is similar to that reported by Kei et al.,^9^ where of 122 neonates, 47.5% had PD type tympanograms and 47.5% had the PU type. Trial probe tone results at 678 Hz indicated a higher occurrence of the AS type curve, which was found in 67.3% of ears. This curve type more difficult to interpret, as the AS type is not typical of normalcy, even though these ears were considered as normal. There was a higher occurrence of PU type tympanograms in the 1000 Hz probe (70.9%). This results was also found in other studies, such as in Kei et al.,^9^ where the PU type occurred in 92% of tympanograms, and in Margolis et al.’s^3^ study.

In this study the 1000 Hz probe provided, statistically, the best results for characterizing tympanogram normalcy, compared to the 226 and 678 Hz probes. This result is similar to those found in the literature; studies by Sutton et al.,^14^ Margolis et al.^3^ and Kei et al.^9^ provide evidence in favor of using the 1000 Hz trial probe tone compared to the 678 Hz probe. Weatherby and Bennett^24^ recommended using the high frequency probe for tympanometry in neonates due to the characteristic differences in middle ear resonance. Keefe et al.^25^ demonstrated that wall movement effects in the external acoustic meatus were minimized by using a high frequency probe tone. Studies done with high frequency tone probes indicate good agreement between tympanometry and the presence of otitis media in neonates.^26^, ^8^

The PU curve type probably characterizes middle ear normalcy, as the neonates that were assessed presented no risk indicators for hearing loss, and TOAEs were detected in all of them. The presence of TOAEs generally suggests normal middle ear function, as there is stimulus transmission to the cochlea and reverse transmission of emissions to the external acoustic meatus.8 Other studies have also considered the PU type as typical of middle ear normalcy.^6^, ^9^, ^27^, ^28^ PD type tympanograms have also been considered as typical of normalcy in the literature.^9^, ^29^ The PD curve, however, rarely is found in high frequency trial probe tones in neonates.^14^ We found no PD recordings in 678 and 1000Hz probes.

No clear reason was found for the occurrence in our sample neonates of other curve types (AS and I) found in high frequency probes, as the middle ears of these neonates were probably normal. Kei et al.^9^ suggest possible reasons, as follows: (i) normal variation within a population; (ii) mild middle ear dysfunction that has not affects TOAEs; (iii) a delay in maturation of the neonatal middle ear; (iv) the probe tone frequency might not have been high enough for some of the neonates; (v) inadequate probe occlusion; and (vi) artifact movement.

The flat type tympanogram is the most common forms of abnormality.^14^ No recording of flat curves was found in the present study.

The probability of finding the same curve type bilaterally in the 110 sample neonates was 70.0% at 226 Hz, 70.9% at 678 Hz and 81.8% at 1000 Hz. This pattern indicated that there was a high probability of a similar curve occurring in both ears of the same neonate. The implications of this finding are that a careful investigation should be made if a curve difference is found between ears; the exam should be repeated and the patient’s clinical history should be carefully evaluated.

In comparing jointly the equal curve types found by using 226 and 1000 Hz probe tones, 20.9% of the neonates were found to present the same curve type at both 226 Hz and 1000 Hz bilaterally. This result suggested that there was a low probability of the same curve type occurring in both ears of the same neonate for both probes.

Qualitative measurements should not be analyzed independently when interpreting the tympanogram; they should be supported by quantitative measurements for increased reliability of curve type interpretation.

There was no ear-related significance effect when using the 226 Hz probe for Vea measurements, although a sex-related significance effects was noted, with higher values in males. Ymt data from TW and TPP presented no ear or sex-related significance effects. TW and TPP measurements using the 1000 Hz probe revealed no ear or sex-related significance effects, and Ymt results showed no ear-related statistical significance, although they did show a sex-related significance effect, with increased values in males.

Quantitative measurements are ancillary parameters that can support the interpretation of tympanometry, increasing the reliability of curve configuration and improving tympanometric diagnosis. Findings should be analyzed jointly to achieve any conclusion about conditions in the middle ear, given the significant variability of quantitative measurements.

Although there was a high occurrence rate of PU type curves when using the 1000 Hz probe, 29.1% of curves were not typical of normalcy (non-PU). Such a high rate of non-PU curves generates an uncertainty index, which complicates tympanometric curve interpretation and a diagnosis based on the tympanometric exam. In such cases quantitative measurements do not support the diagnosis, as in non-PU curves quantitative measurements are not calculated.

As discussed above, the cause of such a high rate of non-PU curves in neonates is poorly understood. Possibly the issue is related not to the measurements themselves, but to the interpretation of tympanograms. Sutton et al.^14^ proposed a simple framework as an alternative form of interpreting tympanograms, using both qualitative and quantitative measurements to indicate tympanogram normalcy based on 678 and 1000 Hz probe tones.

In the current study Sutton et al.’s^14^ interpretation showed that when using the 678 Hz and the 1000Hz probes, the result was nearly 100% normal tympanograms. This high occurrence of normalcy would be expected in the sample neonates. This interpretation is simpler as it offers only two possible options for classifying a tympanogram, which reduces the uncertainty level.

Tympanometry may support auditory diagnosis, but should not be used alone; it should accompany other test results such as BAEP (air and bone transmission), OEAs and otoscopy.

There are few published studies on tympanometry in neonates. Further research is needed to provide additional data for comparison purposes and to increase the reliability of curve interpretation and result validation.

## CONCLUSION

Our data suggest that the 1000 Hz probe had statistically the best results in characterizing tympanograms according to normalcy. The results demonstrate that qualitative and quantitative measurements should now be assessed singly when making the diagnosis, but should rather be interpreted jointly. The variability of findings in neonates requires an ample interpretation of findings to establish the validity of conclusions. The assessment method proposed by Sutton et al.’s^14^ protocol efficiently reduced the variability of tympanometric measurements, increased diagnostic reliability. Our data may be used as normal parameters in tympanometric diagnosis.
